# A World of Viruses Nested within Parasites: Unraveling Viral Diversity within Parasitic Flatworms (Platyhelminthes)

**DOI:** 10.1128/spectrum.00138-22

**Published:** 2022-05-10

**Authors:** Nolwenn M. Dheilly, Pierrick Lucas, Yannick Blanchard, Karyna Rosario

**Affiliations:** a ANSES, Agence Nationale de Sécurité Sanitaire de l’Alimentation, de l’Environnement et du Travail, Laboratoire de Ploufragan-Plouzané, Unité Génétique Virale de Biosécurité, Ploufragan, France; b ANSES Animal Health Laboratory, UMR Virology, ANSES/INRAE/ENVA, Maisons-Alfort, France; c College of Marine Sciences, University of South Florida, Saint Petersburg, Florida, USA; Changchun Veterinary Research Institute

**Keywords:** cestodes, trematodes, bunyavirus, evolution, flatworm, host shift, neodermatan, parasite, rhabdovirus, virome

## Abstract

Because parasites have an inextricable relationship with their host, they have the potential to serve as viral reservoirs or facilitate virus host shifts. And yet, little is known about viruses infecting parasitic hosts except for blood-feeding arthropods that are well-known vectors of zoonotic viruses. Herein, we uncovered viruses of flatworms (phylum Platyhelminthes, group Neodermata) that specialize in parasitizing vertebrates and their ancestral free-living relatives. We discovered 115 novel viral sequences, including 1 in Macrostomorpha, 5 in Polycladida, 44 in Tricladida, 1 in Monogenea, 15 in Cestoda, and 49 in Trematoda, through data mining. The majority of newly identified viruses constitute novel families or genera. Phylogenetic analyses show that the virome of flatworms changed dramatically during the transition of neodermatans to a parasitic lifestyle. Most Neodermata viruses seem to codiversify with their host, with the exception of rhabdoviruses, which may switch hosts more often, based on phylogenetic relationships. Neodermata rhabdoviruses also have a position ancestral to vertebrate-associated rhabdo viruses, including lyssaviruses, suggesting that vertebrate-associated rhabdoviruses emerged from a flatworm rhabdovirus in a parasitized host. This study reveals an extensive diversity of viruses in Platyhelminthes and highlights the need to evaluate the role of viral infection in flatworm-associated diseases.

**IMPORTANCE** Little is known about the diversity of parasite-associated viruses and how these viruses may impact parasite fitness, parasite-host interactions, and virus evolution. The discovery of over a hundred viruses associated with a range of free-living and parasitic flatworms, including parasites of economic and clinical relevance, allowed us to compare the viromes of flatworms with contrasting lifestyles. The results suggest that flatworms acquired novel viruses after their transition to a parasitic lifestyle and highlight the possibility that they acquired viruses from their hosts and vice versa. An interesting example is the discovery of flatworm rhabdoviruses that have a position ancestral to rabies viruses and other vertebrate-associated rhabdoviruses, demonstrating that flatworm-associated viruses have emerged in a vertebrate host at least once in history. Therefore, parasitic flatworms may play a role in virus diversity and emergence. The roles that parasite-infecting viruses play in parasite-associated diseases remain to be investigated.

## INTRODUCTION

Over the past few years, high-throughput sequencing technologies have expanded the virosphere well beyond pathogenic viruses and/or viruses that can be cultured. Therefore, the known taxonomy and understanding of virus evolution have increased dramatically (1). One such breakthrough is the finding that the diversity of plant- and vertebrate-infecting RNA viruses is nested within the diversity of viruses that infect arthropods (2), suggesting that these invertebrates have played a role as viral reservoirs and facilitated host shifts (2, 3). Despite these advances, our current knowledge of the global RNA virome remains strongly biased, with most sequencing efforts focusing on plants, chordates, arthropods, and to a lesser extent, nematodes and mollusks. Sampling viruses from a larger diversity of eukaryotic hosts should lead to new and improved evolutionary scenarios. Here, we investigated viruses found in parasitic flatworms and their free-living relatives to provide an initial assessment of the viral diversity associated with Platyhelminthes with contrasting lifestyles.

**FIG 1:**
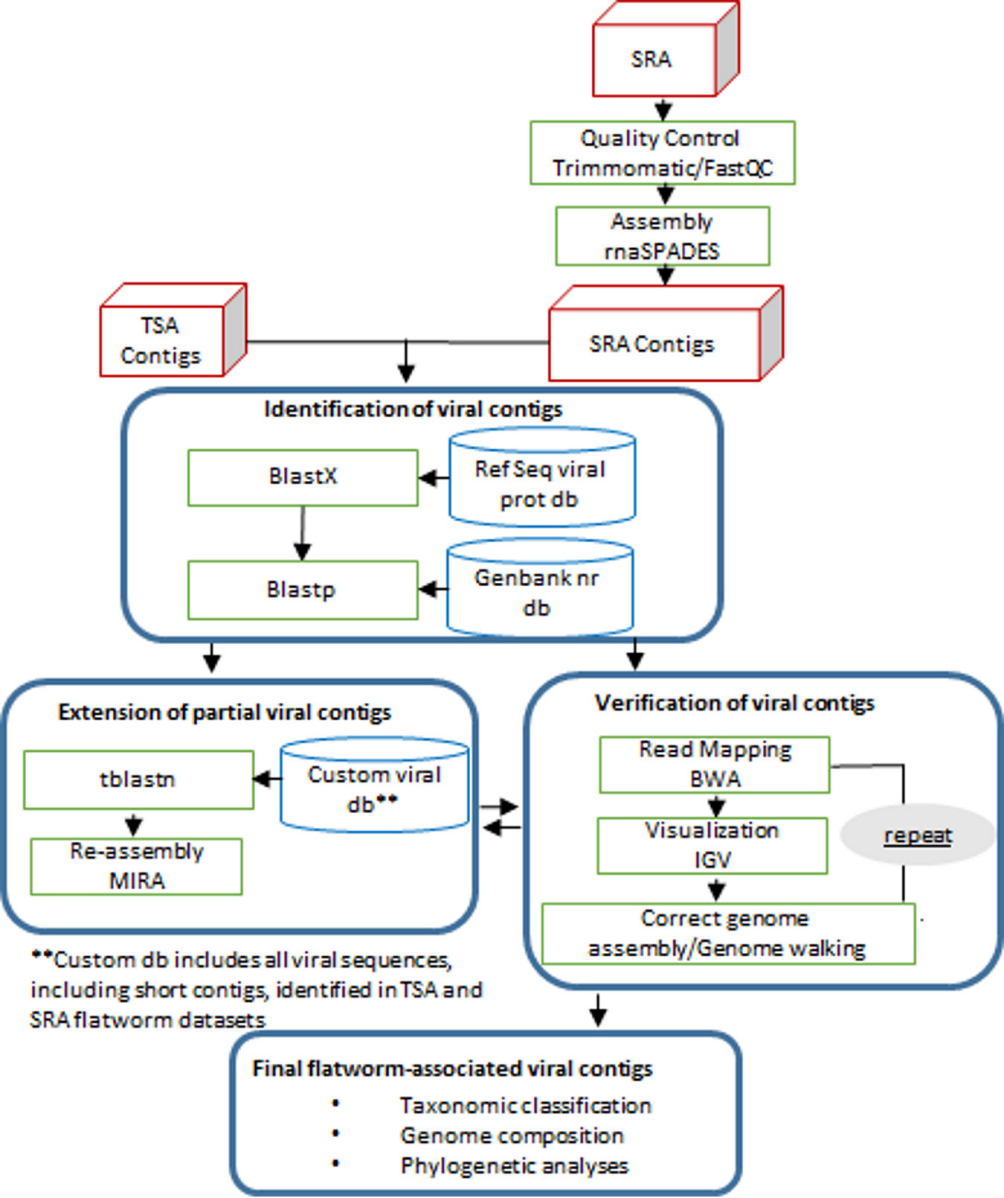
Workflow used for the identification of viral contigs, extension, control of assembly quality, and taxonomic classification.

Platyhelminthes, also known as flatworms, constitute a diverse phylum estimated to contain up to 100,000 species with diverse body plans, lifestyles, and ecological roles (4, 5). The majority of Platyhelminthes are classified within the Rhabditophora subphylum. Ancestral members of this subphylum have a free-living lifestyle, including Tricladida species used for cellular biology research investigating stem cells, aging, tissue regeneration, and homeostasis (Fig. 1) (6–8). On the other hand, the superclass Neodermata, representing more than half of Platyhelminthes biodiversity, groups endoparasitic trematodes (Digenea and Aspidogastrea) and tapeworms (Cestoda) and ectoparasitic monogeneans (Polyopisthocotylea and Monopisthocotylea) (9). Here, we refer to flatworms outside Neodermata as “free living” to distinguish this group with strict parasitic lifestyles. However, note that there are some “non-free living” flatworms outside Neodermata.

Neodermata parasites have contrasting life cycles and are economically relevant pathogens. While monogeneans typically have a single vertebrate fish host, trematodes and tapeworms have complex life cycles that involve a range of vertebrates as definitive hosts, invertebrate intermediate hosts (typically mollusks and crustaceans, but also cnidarians or polychaetes), and sometimes, a second intermediate host (mollusks, crustaceans, plants, fish, or amphibians) (9–12). Neodermata represent a significant economic and health burden given that these parasites infect humans, cattle, and other domesticated animals. For instance, schistosomiasis, caused by several species of trematodes of the genus *Schistosoma*, is the second most important neglected tropical disease after malaria, affecting over 200 million people and causing 200,000 deaths annually worldwide (13). Chronic opisthorchiasis and clonorchiasis, caused by the liver flukes Opisthorchis viverrini, Opisthorchis felineus, and Clonorchis sinensis, have been classified as group I carcinogens by the International Agency for Research on Cancer due to the increased risk of cholangiocarcinoma associated with infection (14, 15). Humans are also subject to infection by several species of tapeworms, including *Taenia* spp., *Echinococcus* spp., and *Diphyllobothrium* spp. (13, 16, 17).

Although our knowledge of flatworm-associated viruses remains very limited, there is evidence indicating that flatworms harbor a diversity of viruses. The very first report of virus-like particles in parasitic Platyhelminthes dates from 1976, with the observation of geometric arrangements of viral particles in parenchymal cells (18). Since then, a few more studies have reported the presence of virus-like particles in monogenean parasites (19, 20) and other flatworms (21–23). The rise of next-generation sequencing technologies has led to the discovery and complete genomic characterization of a single-stranded DNA (ssDNA) virus (24), a large nidovirus (25), and a new family of toti-like viruses (26) in free-living flatworms. Viruses of the order *Bunyavirales* and the family *Nyamiviridae* (order *Mononegavirales*) have been reported from Schistosoma japonicum and a mix of *Taenia* sp. (27). More recently, a comprehensive study investigating the virome of the cestode Schistocephalus solidus demonstrated that parasitic flatworms may be associated with a large diversity of viruses (28). This single species was shown to host multiple species of rhabdovirus, nyamivirus, jingchuvirus, bunya-like virus, and toti-like virus (28).

Here, we screened for the presence of RNA viruses in the transcriptomes of a broad range of flatworm species. By comparing the phylogenetic positions of viruses discovered in ancestral free-living Platyhelminthes and in Neodermata parasites, we explored the impact of the transition to parasitism on the Platyhelminthes virome composition. In addition, we investigated the role of parasite ecology and evolution in virus evolution. When closely related viruses were found, we investigated whether viruses codiversified with their parasitic hosts. Neodermata could provide opportunities for viruses to complete major host shifts across distantly related taxa, given that these parasitic flatworms infect different hosts over the course of their life cycle. Accordingly, we discuss whether parasite viruses have spillover potential based on their evolutionary history.

## RESULTS AND DISCUSSION

### Platyhelminthes harbor a diverse RNA virome.

**FIG 2:**
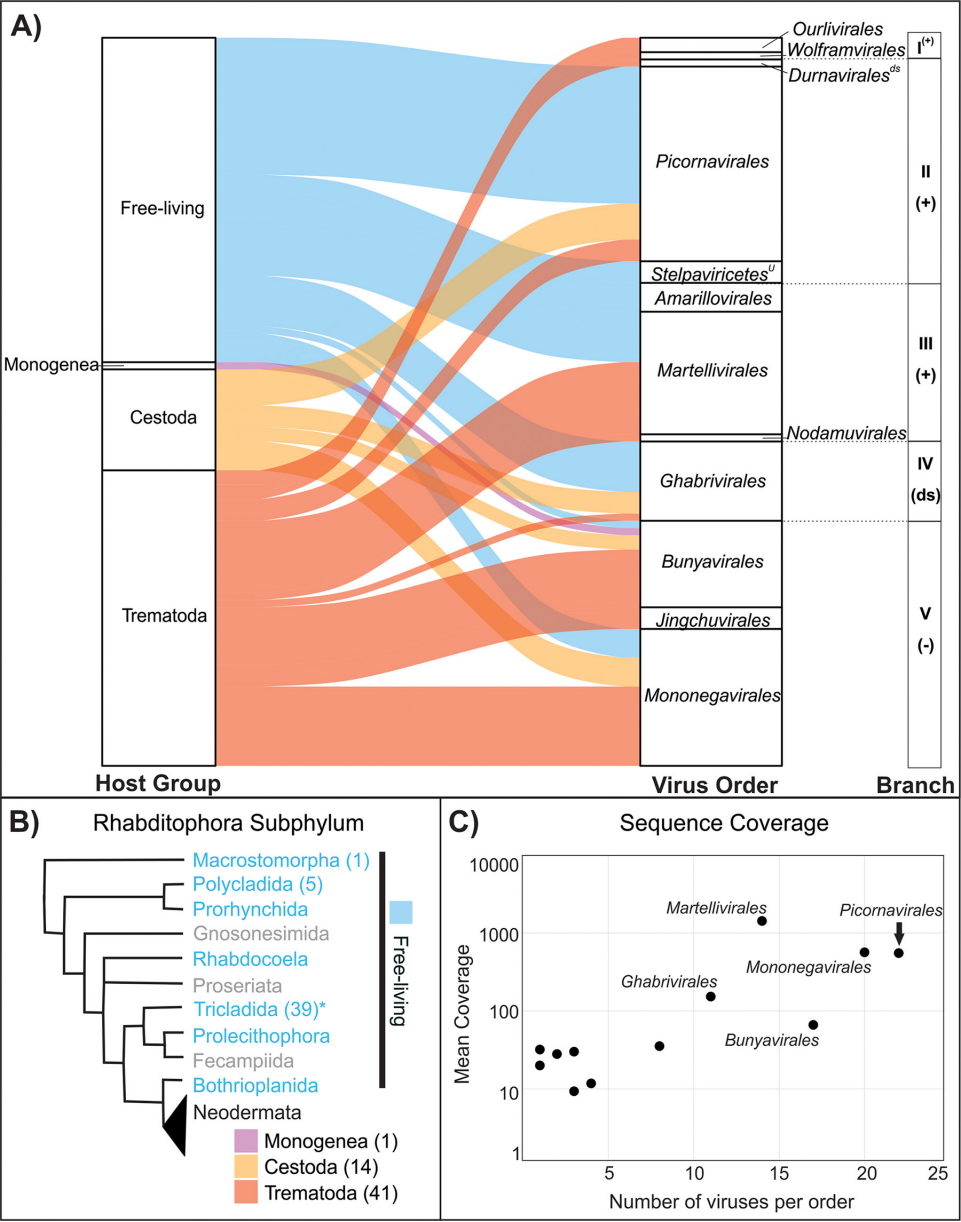
Diversity of viruses discovered in transcriptomic data from 31 species of Platyhelminthes. (A) Alluvial plot depicting the distribution of viral sequences identified within flatworm host groups. Viral sequences representing each of the five major branches of the RNA virosphere were grouped at the order level, with the exception of an unassigned (^U^) order within the class *Stelpaviricetes*. Branches include members of the *Lenarviricota* (I), *Pisuviricota* (II), *Kitrinoviricota* (III), *Duplornaviricota* (IV), and *Negarnaviricota* (IV) phyla and include positive-sense (+) and negative-sense (−) single-stranded RNA viruses and double-stranded RNA viruses (ds). Note that *Durnavirales* is the only order within *Pisuviricota* composed of dsRNA viruses. (B) Schematic cladogram showing relationships among groups of the Rhabditophora subphylum according to Littlewood and Waeschenbach (29). Cladogram colors correspond to the alluvial plot, where groups highlighted in blue font represent free-living Rhabditophora host groups investigated here (*, with the exception of Tricladida, which includes one species, Bdelloura candida, considered to be an ectocommensal) (30). Pink, yellow, and red colors represent strict parasitic groups within Neodermata. Rhabditophora taxa in gray font were not investigated here. Numbers within parentheses indicate the number of viruses detected in a given taxon. (C) Mean coverages for viral sequences identified here summarized at the order level.

We conducted a large-scale survey of Platyhelminthes-associated viruses through data mining of publicly available transcriptomes in the Transcriptome Shotgun Assembly (TSA) and Sequence Read Archive (SRA) databanks (workflow in Fig. 1). In total, 149 data sets, corresponding to 66 flatworm species representing free-living Rhabditophora and parasitic Neodermata, were screened for viruses (Table S1 in the supplemental material). Viruses were successfully detected within data sets from free-living Rhabditophora (45 viruses) and Nematoda, including Trematoda (41 viruses), Cestoda (14 viruses), and Monogenea (1 virus) (Fig. 2). A total of 115 unique sequences with either complete (87 sequences) or partial (28 sequences) protein coding sequence regions were identified, representing 101 novel viruses, with a small minority of viruses with fragmented genomes (Tables S2 and S3). Importantly, the viral sequences investigated were not found in available Platyhelminthes genomes and they encoded proteins without frameshifts, nonsense mutations, or repeat sequences that are common in endogenous viral elements. Therefore, the viruses described here are most likely exogenous functional viruses.

The novel Platyhelminthes-associated virus species were distributed among all five major phyla of RNA viruses and fell within a total of 12 orders, revealing the large diversity of virus taxa found within flatworms (Fig. 2, Table S4). Only two viruses were classified at the genus level (*Nyamiviridae* family, genus *Tapwovirus*) and 34 viruses at the family level, indicating that Platyhelminthes host a unique viral diversity. The majority of viruses were classified within the orders *Picornavirales* (27 viruses), *Mononegavirales* (21 viruses), *Bunyavirales* (17 viruses), *Martellivirales* (14 viruses), *Ghabrivirales* (11 viruses), and *Amarillovirales* (8 viruses) (Fig. 2). Other orders were represented by five members or fewer, including *Wolframvirales*, *Ourlivirales*, *Durnavirale*s, an unassigned order of *Stelpaviricetes*, *Amarillovirales*, *Nodamuvirales*, and *Jingchuvirales*. We observed a positive correlation (Spearman’s rank correlation *r* = 0.75; *P* = 0.0045) between the number of viruses discovered within an order and the mean read coverage of those viruses (Fig. 2C). It is possible that viral taxa with low mean coverage are less represented in flatworms. Alternatively, it is possible that the methodological and sequencing approaches used in transcriptomic studies investigated here did not recover viruses with low abundance, which would suggest that Platyhelminthes host an even greater diversity of viruses than presented herein.

We investigated phylogenetic relationships among Platyhelminthes-associated viral taxa. Viruses within unassigned families of the orders *Picornavirales*, *Martellivirales*, *Ghabrivirales*, *Bunyavirales* and *Jingchuvirales*, as well as viruses within the families *Flaviviridae*, *Nyamiviridae*, and *Rhabdoviridae*, were found within more than one Platyhelminthes species. We used phylogenetic methods to further investigate their relationships to each other and to the known viral diversity. To build phylogenetic trees, we included representatives of previously characterized families, as well as unassigned viruses that showed high sequence similarity to viruses described here. Phylogenetic analyses revealed that viruses of Platyhelminthes often cluster together, separately from other known viruses, providing evidence that they constitute distinct taxa. Note that newly discovered flatworm-associated viruses that were not phylogenetically related to other viruses of Platyhelminthes were not investigated further because the host remains putative. These viruses could be associated with the host diet or with a coinfecting microorganism or could result from contamination during sample processing. Overall, including only taxa for which viruses were found in at least two different Platyhelminthes species and based on a combination of phylogenetic analyses, genome composition analyses, and shared percentages of identity (see below), our data provide evidence for at least seven new families and 18 new genera of Platyhelminthes-specific viruses that will be submitted to ICTV for evaluation (Fig. 2, Fig. S1 to 16, Table S3).

### Previously reported viruses of Neodermata parasites within vertebrate and invertebrate hosts.

In some instances, the newly identified Neodermata-associated viruses clustered closely with vertebrate- and invertebrate-associated viruses previously discovered through metagenomic and metatranscriptomic studies (2, 27, 31). However, upon close inspection, we found transcripts that belong to Platyhelminthes within the original data sets from these reports, indicating that parasites were present at the time of sampling. Specifically, we found transcripts from an unknown trematode (likely from the family Fasciolidae or Dicrocoeliidae) in the data set from a razor shell specimen (SRR3401916) that contained a picorna-like virus (Beihai razor-shell virus 4) and a bunya-like virus (Beihai bunya-like virus 2) closely related to trematode-associated viruses discovered here. The spotted paddle-tail newt (SRR6291293), within which a Neodermata virus-like rhabdovirus was found (Fujian dimarhabdovirus), appeared infected by trematodes known to infect amphibians and nonfish vertebrates (*Mesocoelium* sp. and *Spirometra* sp.). The Wenling sharpspine skate mix (SRR6291349) within which another Neodermata virus-like rhabdovirus was found (Wenling dimarhabdovirus 8) was infected by a cestode from the family Echinobothriidae known to infect Elasmobranchii. Finally, two sample mixes of fish gills (SRR6291357 and SRR6291374) within which more Neodermata virus-like rhabdoviruses were found (Wenling dimarhabdovirus 10 and Beihai dimarhabdovirus 1) contained reads that aligned against monogenean parasite nucleotide sequences, but the low percentage of reads belonging to Platyhelminthes prevented the successful assembly of transcripts. The phylogenetic positions of these viruses and the demonstration that the host organisms were infected by Neodermata parasites at the time of sampling suggest that these viruses could be infecting the parasite rather than the vertebrate or invertebrate host. Additional viruses that were probably associated with tapeworms or flukes but for which we could not conduct the same analysis (either the raw data were not available or samples were processed in a way that eliminated host-associated transcripts) include picornaviruses (fesavirus 3 [32], Pernambuco virus [33], arivirus 2 [34], and blackbird arilivirus [35]) and an additional rhabdovirus (fox fecal rhabdovirus [36]). Future studies investigating the viromes of vertebrates, mollusks, or crustaceans that are either definitive or intermediate hosts of Neodermata parasites need to consider the presence of such stowaway passengers when assigning hosts to newly discovered viruses.

### Neodermata viruses are distinct from viruses of free-living Rhabditophora.

**FIG 3:**
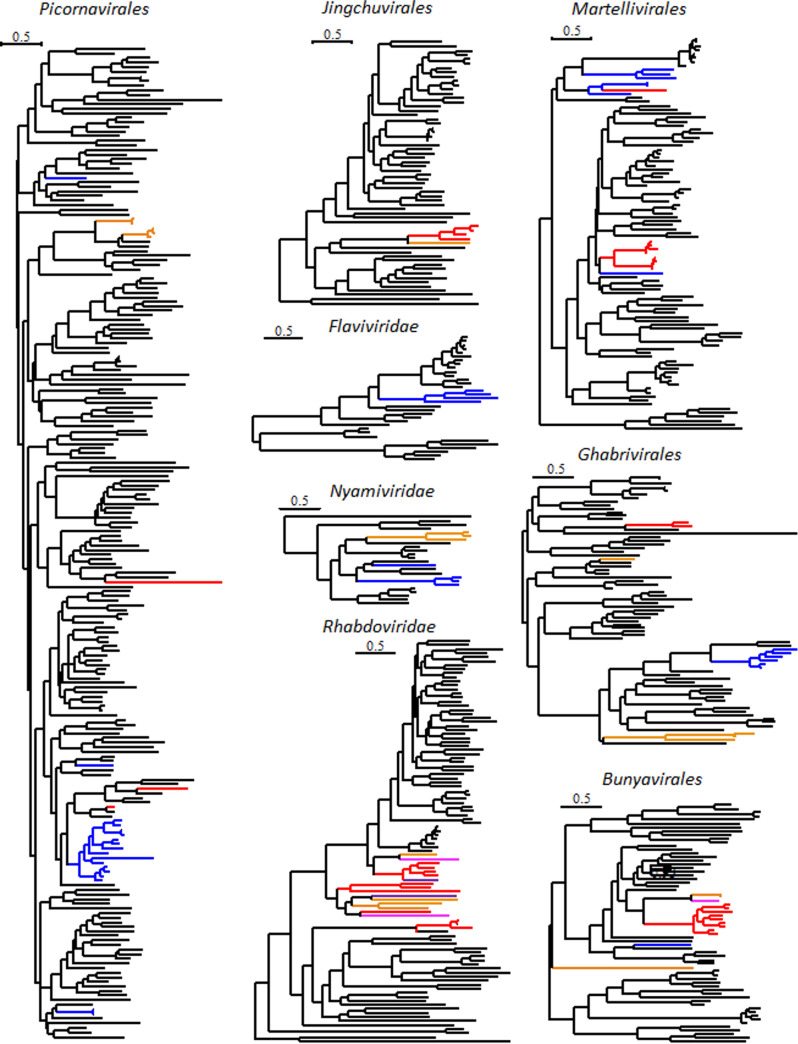
Distinct viruses identified within free-living Rhabditophora and parasitic Neodermata. Phylogenetic trees of the RNA-directed RNA polymerases (RdRPs) of RNA viruses of the orders *Picornavirales*, *Martellivirales*, *Bunyavirales*, *Ghabrivirales*, and *Jingchuvirales* and the families *Flaviviridae*, *Nyamiviridae*, and *Rhabdoviridae*. Viruses of Platyhelminthes included in the trees are color coded (red, Trematoda; orange, Cestoda; pink, Monogenea; blue, Rhabditophora). The trees were inferred in PhyML, and high-resolution annotated trees are available in Fig. S1 to S8.

Differences between free-living and parasitic Platyhelminthes were reflected in the types of viruses they harbored. We discovered a greater diversity of positive-strand single-stranded RNA viruses and double-stranded RNA viruses of the families *Picornavirales* and *Ghabrivirales* in the free-living Rhabditophora than in the parasitic Neodermata (Fig. 2). A similar trend was observed in the only non-free-living flatworm included in the free-living Rhabditophora data set, an ectocommensal named Bdelloura candida. In contrast, Neodermata parasites harbored a greater diversity of negative-strand single-stranded RNA viruses. For example, negative-strand RNA viruses of a novel family within the order *Jingchuvirales* and viruses of the family *Rhabdoviridae*, order *Mononegavirales*, were found exclusively in Neodermata parasites (Fig. 2). When viruses of a given clade were found in both ancestral Platyhelminthes and Neodermata parasites, they clustered separately on phylogenetic trees. This distinct clustering was evident for unassigned viral families of the orders *Picornavirales*, *Bunyavirales*, and *Ghabrivirales* (Fig. 3). There were only two cases where viruses of Neodermata and Rhabditophora clustered together on the phylogenetic tree. Within the family *Nyamiviridae*, viruses of tapeworms clustered closely together within the genus *Tapwovirus*, whereas viruses of Rhabditophora were more closely related to viruses of the genus *Berhavirus* (Fig. 3). Within the order *Martellivirales*, the Psilosi virus found in the trematode *Psilotrema simillimum* clustered closely with viruses of Rhabditophora (Planaria torva and Schmidtea mediterranea), whereas the Provittati virus of the free-living worm *Prostheceraeus vittatus* clustered most closely with viruses of liver flukes. And yet, the RNA-dependent RNA polymerase (RdRP) proteins of these viruses showed maximums of 36% and 45% amino acid identity to their closest relatives, suggesting that they belong to different taxa. More sampling is needed to help resolve the phylogeny of Platyhelminthes. Nevertheless, our findings indicate that the transition of a protoneodermatan worm from free living to parasitism over 500 mega-annum (Ma) ago (37) impacted virus evolution.

### Viruses of Neodermata codiversify with their parasitic hosts.

**FIG 4:**
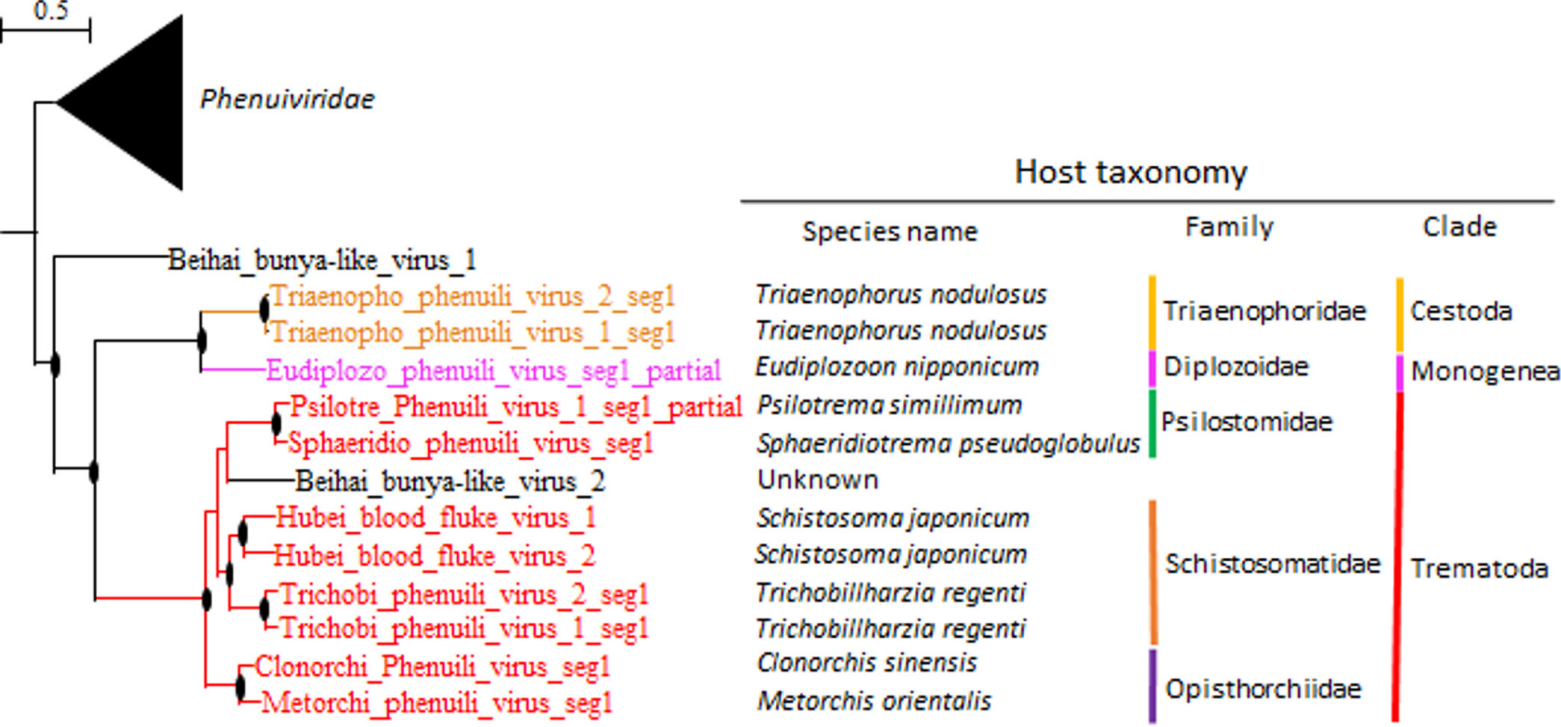
Neodermata viruses of the order Bunyavirales codiversify with their parasitic hosts. The figure provides a phylogenetic tree of the RNA-directed RNA polymerases (RdRPs) of RNA viruses of the order Bunyavirales found in Neodermata parasites and closely related viruses of the family *Phenuiviridae.* Viruses of Platyhelminthes included in the trees are color coded (red, Trematoda; orange, Cestoda; blue, Rhabditophora). The tree was inferred in PhyML using the LG substitution model. Branch points indicate that the results of the Shimodaira-Hasegawa branch test were >0.9. The species name, family, and order of the hosts of identified viruses are provided next to the branches.

Viruses of Neodermata, with the exception of rhabdoviruses (see below), often clustered separately based on their parasitic host’s phylogenetic relationships, suggesting a close association between parasitic hosts and their viruses. Within the order *Ghabrivirales*, viruses of cestodes and trematodes are found on distinct branches and constitute two novel proposed families, suggesting distinct evolutionary origins before diversification within their parasitic hosts (Fig. 3). Within the order *Jingchuvirales*, Neodermata viruses showed a maximum of 19% amino acid identity and clustered separately from all other viruses, suggesting that they constitute a novel family (Fig. 3). Among these, the cestode-associated virus Schistocephalus solidus jingchuvirus (SsJV) had only 23 to 29% amino acid identity with jingchuviruses of trematodes, indicating that they belong to two distinct genera within the same family. Similarly, viruses of the order *Bunyavirales* associated with trematodes clustered separately (with 19 to 23% identity) from the viruses associated with the monogenean Eudiplozoon nipponicum and the cestode Triaenophorus nodulosus (Fig. 4). Those two viruses showed 40% amino acid identity, while Bunya-like viruses of trematodes clustered further, depending on the parasite’s family, with 57 to 71% identity when the hosts belonged to the same family but 37 to 47% identity when the hosts belonged to different parasite families. Viruses associated with the Schistosomatidae (Schistosoma japonicum and Trichobillharzia regenti) clustered together, separately from viruses associated with the liver flukes of the family Opisthorchiidae (Metorchis orientalis and Clonorchis sinensis) that clustered together, and separately from viruses associated with the Psilostomidae (Psilotrema simillimum and Sphaeridiotrema pseudoglobulus). Past experimental investigations on the transmission mode of Schistocephalus solidus viruses revealed that most viruses, including SsJV and a Bunya-like virus, are vertically transmitted from parents to offspring (28). The codiversification of jingchuviruses and Bunya-like viruses with their cestode and trematode hosts supports this finding and indicates that vertical transmission may be common within these new virus taxa.

### Rhabdoviruses exemplify the potential role of parasites in virus evolution.

**FIG 5:**
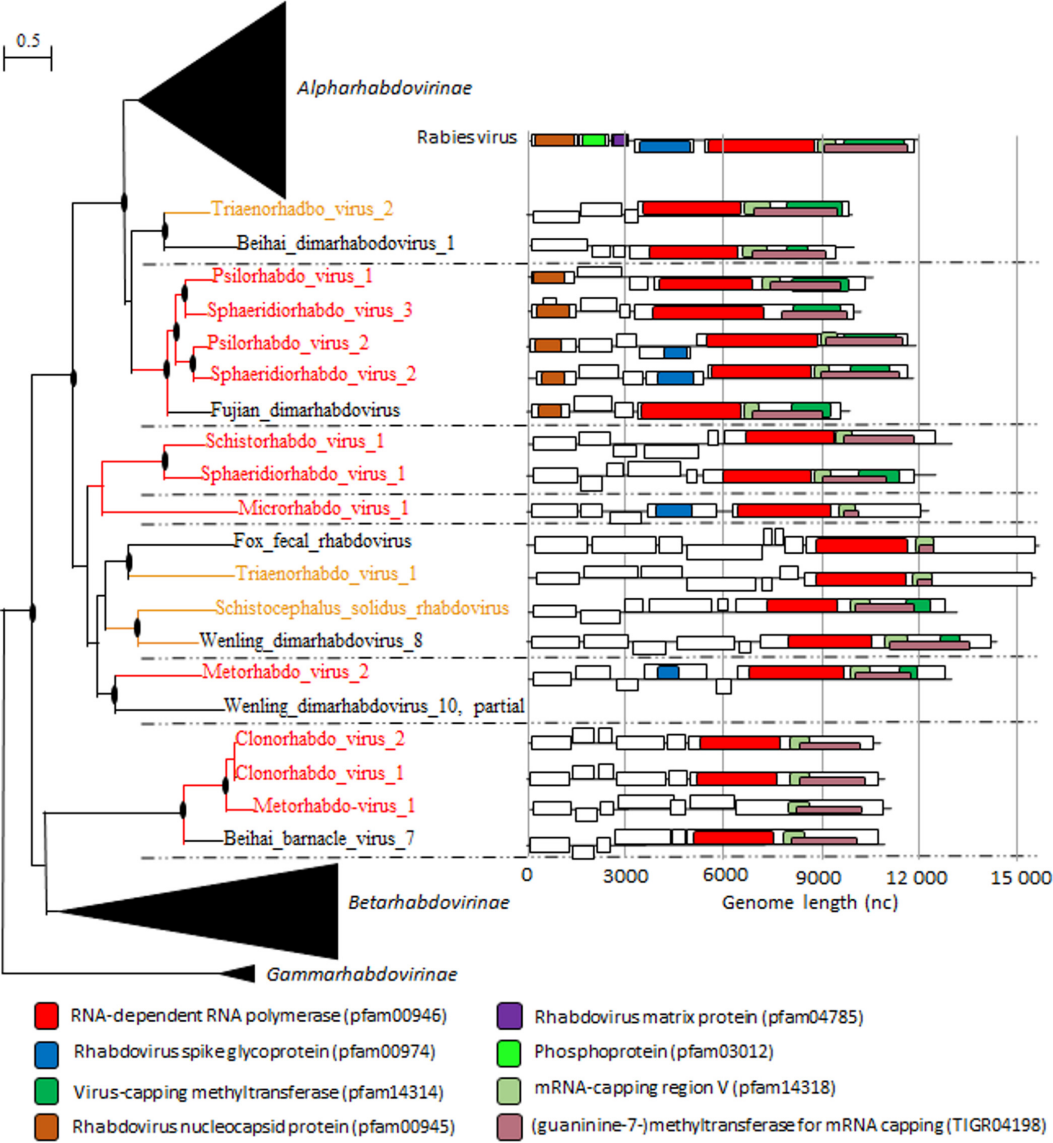
Neodermata viruses of the family *Rhabdoviridae*. Left, phylogenetic trees of the RNA-directed RNA polymerases (RdRPs) of RNA viruses of the family *Rhabdoviridae*. Viruses of Platyhelminthes included in the trees are color coded (red, Trematoda; orange, Cestoda). The dotted lines delineate different taxa. The tree was inferred in PhyML using the LG substitution model. Branch points indicate that the results of the Shimodaira-Hasegawa branch test were >0.9. Right, genome organization of Neodermata viruses of the family *Rhabdoviridae*. Boxes represent putative genes, with color coding identified below. Black lines indicate noncoding regions.

Parasites have intimate relationships with their hosts, and they can infect different host individuals or species over the course of their life cycles, two factors that can facilitate virus transmission and spillover (3). The phylogenetic positions of the diverse novel rhabdoviruses of neodermatan parasites suggest that these viruses switch hosts often and can at times emerge in their hosts. Even though Neodermata-associated rhabdoviruses mostly clustered together, we did not observe a distinct codiversification with their hosts (Fig. 5). Indeed, in line with the demarcation criteria for other rhabdoviruses of a 30% amino acid identity threshold at the subfamily level, a 50% amino acid identity threshold at the genus level, and an 80% amino acid identity threshold at the species level, our analysis suggests that the 13 novel rhabdoviruses are distinct species that belong to a minimum of seven distinct genera within two distinct subfamilies (Fig. 5, Fig. S8). There was no obvious clustering of viruses of cestodes and trematodes, indicating that virus host shifts occurred on multiple occasions. Hahn et al. (28) recently showed that the rhabdovirus SsRV1 is excreted by adult Schistocephalus solidus worms and is transmitted to parasitized hosts. If this characteristic is conserved among Neodermata-associated rhabdoviruses, it would provide an avenue for rhabdovirus host switching between parasites coinfecting the same hosts and between parasites and their intermediate and definitive hosts.

The genomes of rhabdoviruses of cestodes and trematodes exhibited extensive diversity in genome length (from 9,963 nt to 15,554 nt). As expected, this genome length variation was associated with variation in the number of predicted genes. The rhabdovirus genome is normally composed of a minimum of five open reading frames (ORFs) encoding five structural proteins—the nucleoprotein (N), the polymerase-associated phosphoprotein (P), the matrix protein (M), the glycoprotein (G), and the RdRP (L). And yet, five of the rhabdoviruses of Neodermata encoded only four proteins, with the loss of the spike glycoprotein. Other Neodermata rhabdovirus genomes contained up to three additional small ORFs between G and L, encoding putative proteins.

When looking at a broader resolution, all the known diversity of alpharhabdoviruses and betarhabdoviruses is nested within the diversity of viruses of Platyhelminthes, with gammarhabdoviruses of fish maintaining a position ancestral to all rhabdoviruses. This is notable considering that alpharhabdoviruses contain vertebrate and vector-borne rhabdoviruses and betarhabdoviruses encompass plant and arthropod viruses (38). A parsimonious explanation for this phylogenetic distribution would be that an ancestral Neodermata parasite initially acquired a rhabdovirus from its fish host. Then, over the course of Neodermata diversification and an increasing range of intermediate and definitive hosts, these viruses have switched hosts again, giving rise to the diversity of alpharhabdoviruses and betarhabdoviruses known to date. Most specifically, our phylogenetic analysis shows that rhabdoviruses of Neodermata parasites are close ancestors to lyssaviruses, indicating that a parasite-associated rhabdovirus emerged in a parasitized vertebrate host and became the ancestral lyssavirus. Clearly, throughout their evolutionary history, rhabdoviruses have maintained the ability to host switch frequently (39). Host switching would explain why rhabdoviruses often emerge, or reemerge, as zoonotic and epizootic viral diseases (40) and the diversity of host associations within the *Alpharhabdovirinae* subfamily (39). Our analysis indicates that Neodermata virus-associated rhabdoviruses should be considered a potential source of viral emergence.

### Role of viruses in parasite infection and perspectives.

Much remains to be learned about parasitic-flatworm viruses and their role in parasite ecology and evolution. Which viruses of Neodermata can infect parasitized vertebrate and invertebrate hosts? Can these viruses be responsible for symptoms and associated pathologies that have been attributed to the parasitic flatworms? Does viral infection have a deleterious effect on parasite fitness? Alternatively, can viruses increase parasitic-flatworm reproduction and transmission? What is the effect of coinfection by a parasite and its associated virus on host immune response? How do viruses contribute to host–parasitic-flatworm coevolution?

Here, we took advantage of publicly available transcriptomic data to identify viruses. However, transcriptomic data generated from parasitic flatworms or their hosts could also be used to gain additional information on virus prevalence, transmission to the host, or cellular location. For instance, we used a series of transcriptomic data sets (41, 42) to investigate a novel dicistro-like virus named Schmimed virus 1. The transcriptomic data revealed Schmimed virus 1 neurotropism and the role of the Hippo pathway in controlling virus replication within its planarian host (Fig. S17).

Gaining knowledge about Neodermata viral infections would allow us to understand how viruses may affect parasite-host interactions. Viruses could play a role in parasitic flatworm infections, as exemplified by tripartite interactions in other systems. In the parasitoid wasp Leptopilina boulardi, the Leptopilina boulardi filamentous virus (LbFV) manipulates the parasitic wasp’s behavior to increase hyperparasitism—forcing the wasp to lay its eggs in already parasitized hosts—and increase egg load to increase its horizontal transmission (43–45). Another parasitoid wasp, Dinocampus coccinellae, transmits a neurotropic RNA virus to its coccinellid host to manipulate the host behavior and force it to protect the parasite progeny (46). In *Leishmania* (*Viannia*), leishmania RNA virus 1 (LRV1) is excreted within exosomes and exacerbates *Leishmania*’s pathogenicity by causing a hyperinflammatory response and metastatic secondary lesions, known as mucocutaneous leishmaniasis (47, 48). In another protozoan, Trichomonas vaginalis, the trichomonavirus (Trichomonas vaginalis virus [TVV])-induced proinflammatory innate immune response is amplified upon antiparasitic treatment due to the release of viruses by dying parasites (49, 50). Clearly, the potential role of viruses in Neodermata pathogenicity and in symptoms associated with antiparasitic treatments is broad and merits in-depth investigation (51–53). Depending on the nature of virus-parasite interactions and impact on the parasitized host, viruses may be used as biocontrol agents to reduce parasite population size or they may be targets of new vaccines or antiviral treatments to reduce parasite pathogenicity. Characterizing viruses of parasites also offers new opportunities in functional genomics. It has been proposed that viruses of parasites could be used to produce pseudotyped viruses with high specificity to the target parasite species to produce stable lines of transgenic parasites (54).

## MATERIALS AND METHODS

### Building a library of Platyhelminthes transcriptomes.

To discover viruses of Platyhelminthes, we downloaded from the Transcriptome Shotgun Assembly (TSA) sequence database all 45 assembled transcriptomes available, corresponding to 38 flatworm species (Table S1). In addition, we downloaded 104 Sequence Read Archive (SRA) files (Table S1) from trematodes and cestodes for which no assembled transcriptome was available, which allowed us to process data for 28 additional species. All SRA data sets were assembled in-house. For this purpose, raw sequences were trimmed for quality and adapter removal using Trimmomatic version 0.36.0 (55) with default parameters. Sequence quality after trimming was verified with FastQC version 0.11.5 (56), and sequences were assembled with rnaSPAdes as implemented in the SPAdes assembler version 3.11.1 (57).

### Searching for viruses in Platyhelminthes transcriptomes.

Initially, viral contigs larger than 500 bp were discovered in TSA and assembled SRA data by comparing (BLASTx, E value of <10^−10^) against a viral protein database containing sequences from the NCBI Reference Sequence database (RefSeq release number 93, https://www.ncbi.nlm.nih.gov/refseq/). All putative viral transcripts were then translated into proteins to conduct reciprocal BLASTp against GenBank nonredundant (nr) protein database 244, released on 25 June 2021, and confirm virus discovery (Table S2). When partial viral genomes were identified in TSA data sets, corresponding raw reads were downloaded from SRA and reassembled in-house as described above. Next, viral contigs within in-house-reassembled transcriptomes were identified through BLASTx against a viral protein database containing flatworm-associated viral sequences initially discovered from TSA and SRA data and closely related sequences. Reassembly efforts and BLAST searches allowed us to complete or extend the length of partial genome sequences by obtaining longer contigs from in-house assemblies and/or identifying smaller contigs to be used for scaffolding. Viral genome structures and sequences were validated and eventually corrected by mapping all reads and assembled contigs against the newly identified viral genomes as references using Burroughs-Wheeler Aligner (BWA) (version 0.7.8) (58) and were visualized using Integrative Genomics Viewer (IGV) (59, 60). When a given genome structure appeared imperfect, an additional assembly, complementary to the initial SPAdes assembly, was obtained with MIRA (61). Rounds of manual genome sequence correction, read alignment, and visualization were conducted. The complete list of viral sequences identified within this study is provided in Table S3. Final genome annotations were obtained through BLASTx against RefSeq release number 207, updated on 12 July 2021, and BLASTp against GenBank nr protein database 248, updated on 21 February 2022. The distribution of final viral sequences among the investigated flatworm hosts was visualized using an alluvial plot prepared in R version 4.0.5 (https://cran.r-project.org/web/packages/ggalluvial/vignettes/ggalluvial.html).

### Virus genome characterization and phylogenetic analyses.

Open reading frame predictions were obtained using Translate on Expasy and from alignments with related reference virus genomes. Annotation of domains was extracted from comparisons against the Conserved Domain Database (CDD) as implemented by BLASTp against the nr protein database. Initial supergroup assignation was determined from best BLAST matches. Viral RNA-dependent RNA polymerase (RdRP) sequences were aligned, using the E-INS-I algorithm implemented in the program MAFFT (version 7) (62), to representative sequences of all viral families and genera ratified by the ICTV, as well as additional newly described taxa from recent metatranscriptomic studies (2, 27, 31, 63). Ambiguously aligned regions were removed using TrimAl (version 1.2) (64). For each data set, the best-fit model of amino acid substitution was determined using Smart Model Selection (SMS) as implemented in PhyML (version 3.0) (65). Phylogenetic trees were then inferred using the maximum-likelihood method implemented in PhyML (version 3.0) (65) using the best-fit model and best of Nearest-Neighbor Interchange (NNI) and Subtree Pruning and Regrafting (SPR) branch swapping. Support for nodes on the trees was assessed using an approximate likelihood ratio test (aLRT) with the Shimodaira-Hasegawa-like procedure. Viruses were tentatively taxonomically classified whenever possible based on their phylogenetic positions, pairwise sequence identities, pairwise sequence comparisons (66), and/or species demarcation thresholds set by the ICTV (Table S3).

### Searching for parasite transcripts in the source data of suspected parasite viruses.

Our phylogenetic analysis revealed that some viruses associated with nonparasitic hosts were very closely related to viruses of Neodermata parasites. To determine whether the detection of these previously reported viral sequences resulted from contamination due to parasitized hosts at the time of sampling, we investigated the presence of parasite sequences. To do this, SRA data sets SRR6291374, SRR6291293, SRR6291349, SRR6291357, and SRR3401916 were downloaded and assembled as described above using SPAdes. Transcript annotation was conducted using MegaBLAST against GenBank to identify transcripts of Neodermata. In addition, the taxonomic composition from each data set was assessed by comparing 1% of the reads against GenBank. The taxonomic composition was visualized using the Krona chart (67). The presence of Neodermata was confirmed when (i) reads aligned against a parasite whose ecology matched with the sample and (ii) transcripts had significant BLAST matches to Neodermata parasite proteins (i.e., cytochrome c oxidase, transcription elongation factor, heat shock protein 70 [HSP70], or ATPase).

### Data availability.

Viral sequences are available under GenBank accession numbers BK059652 to BK059766 as indicated in Table S3.
